# Fbw7 Inhibits the Progression of Activated B-Cell Like Diffuse Large B-Cell Lymphoma by Targeting the Positive Feedback Loop of the LDHA/lactate/miR-223 Axis

**DOI:** 10.3389/fonc.2022.842356

**Published:** 2022-03-10

**Authors:** Su Yao, Tairan Guo, Fen Zhang, Yu Chen, Fangping Xu, Donglan Luo, Xinlan Luo, Danyi Lin, Wendan Chen, Zhi Li, Yanhui Liu

**Affiliations:** Department of Pathology, Guangdong Provincial People’s Hospital, Guangdong Academy of Medical Sciences, Guangzhou, China

**Keywords:** Fbw7, ubiquitination, LDHA, Lactate, miR-223, activated B-cell, diffuse large B-cell lymphoma

## Abstract

**Background:**

F-box and WD repeat domain-containing 7 (Fbw7) is well known as a tumor suppressor and ubiquitin ligase which targets a variety of oncogenic proteins for proteolysis. We previously reported that Fbw7 promotes apoptosis in diffuse large B-cell lymphoma (DLBCL) through Fbw7-mediated ubiquitination of Stat3. This study aimed to identify the mechanism of Fbw7-mediated aerobic glycolysis reprogramming in DLBCL.

**Methods:**

Expression levels of Fbw7 and Lactate Dehydrogenase A (LDHA) in human DLBCL samples were evaluated by immunohistochemistry. Crosstalk between Fbw7 and LDHA signaling was analyzed by co-immunoprecipitation, ubiquitination assay, western blotting and mRNA quanlitative analyses. *In vitro* and *in vivo* experiments were used to assess the effect of the Fbw7-mediated LDHA/lactate/miR-223 axis on DLBCL cells growth.

**Results:**

Fbw7 could interact with LDHA to trigger its ubiquitination and degradation. Inversely, lactate negatively regulated Fbw7 *via* trigging the expression of miR-223, which targeted Fbw7 3’-UTR to inhibit its expression. *In vivo* and *in vitro* experiments revealed that miR-223 promoted tumor growth and that the effects of miR-223 on tumor growth were primarily related to the inhibition of Fbw7-mediated LDHA’s ubiquitination.

**Conclusions:**

We demonstrated that the ubiquitin-ligase Fbw7 played a key role in LDHA-related aerobic glycolysis reprogramming in DLBCL. Our study uncovers a negative functional loop consisting of a Fbw7-mediated LDHA/lactate/miR-223 axis, which may support the future ABC-DLBCL therapy by targeting LDHA-related inhibition.

## Introduction

Diffuse large B-cell lymphoma (DLBCL) is the most common subtype of non-Hodgkin lymphoma (NHL) which derived from mature B cells and accounts for 30% to 35% of all nodal lymphomas (1, 2). Based on generation sequencing and cell-of-origin, DLBCL could be stratified into two molecular subtypes, the activated B cell (ABC) type and the germinal center B cell (GCB) type. ABC-DLBCL is related with tumor aggressive growth and substantially worse outcomes (3, 4). It is also characterized with increased drug resistance and constitutive activation of NF-kB (5). However, the molecular characterization of ABC-DLBCL-specific malignant phenotype remains elusive (6, 7).

The reprogramming of glucose metabolism is a critical aspect of tumor development and progression. To make a sufficient supplementary of intermediary metabolites for the ATP generation and synthesis of new biomass, tumors often rewrite their metabolism program by activating a group of glycolysis-related enzymes (8). An increased rate of glycolysis enables the diversion of metabolites into pathways that promote the biosynthesis of organelles and macromolecules required for tumor growth and resistance to chemotherapy (9–11). Serum lactate dehydrogenase (LDH) is a key marker for aggressive non-Hodgkin lymphoma (NHL) and is one of the factors listed in the International Prognostic index (IPI) (12, 13). High LDH activity was reported following treatment with hematopoietic growth factors and intensive chemotherapy regimens (14, 15). LDH is a family of NAD+-dependent enzymes. It is comprised of two major subunits, LDHA and LDHB, which can assemble into five different isoenzymes (LDH1, LDH2, LDH3, LDH4, and LDH5) (16–18). LDHA, which is encoded by the gene LDHA, is the predominant form in skeletal muscle and mostly involved in anaerobic metabolism with a higher affinity for pyruvate. In tumor anaerobic glycolysis, LDHA preferentially catalyzing pyruvate to lactate and which is undisputed known as a vital checkpoint (19). However, the LDHA-related tumor metabolism reprogramming in DLBCL remains unclear.

F-box and WD repeat domain-containing 7 (Fbw7) is a ubiquitin ligase that acts an important role in cancer by targeting several key proteins for proteolysis (20–25). In previous study, we demonstrated that Fbw7 promotes apoptosis *via* Fbw7-mediated ubiquitination of Stat3 in ABC-DLBCL (26). However, the role of Fbw7 in tumor metabolism is still unknown. Here, we demonstrated that Fbw7 plays a key role in LDHA-associated metabolism in ABC DLBCL. Fbw7 interacts with LDHA which results in its ubiquitination and degradation. Moreover, LDHA-associated lactic acid accumulation in ABC DLBCL is critical for tumor aggressive progression. Interestingly, we confirmed that lactate negatively regulates Fbw7 by inducing the expression of miR-223. MiR-223 targets the Fbw7 3’-UTR thus inhibiting its expression. *In vivo* and *in vitro* studies showed that miR-223 promotes tumor growth and these effects are associated with the negative regulatory effects of Fbw7-mediated ubiquitination of LDHA.

Taken together, these results unraveled underlying molecular mechanism of Fbw7 in reprogramming glucose metabolism. The LDHA/lactate/miR-223 axis promotes the progression of ABC DLBCL and is a promising therapeutic target. Our study reveals a negative functional loop, the Fbw7-mediated LDHA/lactate/miR-223 axis, and our findings may constitute a promising therapeutic target for ABC-DLBCL patients.

## Materials And Methods

### Cell Culture

The ABC-DLBCL cell lines OCI-LY-3, SU-DHL-2, U2932, OCI-LY-10 and HEK293 were cultured in RPMI-1640 supplemented with 10% FBS (Gibco, Carlsbad, CA, USA) and maintained in incubators with 5% CO_2_ at 37°C. The cell lines were confirmed by ATCC STR database.

### Cell Transfection

Plasmids encoding Flag-Fbw7, His-LDHA and HA-Ubiquitin were constructed by cloning PCR amplified into pcDNA3.1. LDHA siRNA target sequence was 5’-GUUCAUCAUUCCCAACAUUTT-3’ (siRNA1) and 5’- AAUGUUGGGAAUGAUGAACTT-3’ (siRNA2). LDHA siRNA and siRNA-control (siRNA-NC), miR-223 inhibitor and mimic were purchased from RIBOBIO (Guangzhou, China). The constructs were transfected using Lipofectamine 3000 according to the manufacture (Invitrogen, Carlsbad, CA, USA).

### Metabolism Analysis

The Lactate dehydrogenase (LDH) assay kit (C0016, Beyotime Biotechnology) and L-Lactate Assay Kit II (1200051002, Eton Bioscience) were used to measure intracellular LDH and lactate levels, respectively, in DLBCL cells according to the manufacturer’s protocols. ATP generation was detected using an ATP detection assay kit (S0026, Beyotime Biotechnology).

### Tissue Samples and IHC

Thirty-two formalin-fixed paraffin-embedded (FFPE) DLBCL tissues were collected at the Guangdong Provincial People’s Hospital. And written informed consent was obtained from all of the patients and this study was approved by the Biomedical Research Ethics Committee of Guangdong Provincial People’s Hospital. And patients with detail clinicopathological characteristics are rendered in **Supplementary Table S1**. For immunohistochemistry (IHC) detection, Paraffin embedded tissues were sectioned at 4-µm thickness, microwaved in Na citrate buffer, pH 6, for 10min and following primary antibody. Secondary antibody was biotinylated, and then sections were developed with diaminobenzidine. Staining scores were estimated by two pathologists in a blinded fashion. A quick scoring criteria was: 0 (no staining), 1 (weak staining), 2 (intermediate staining), and 3 (strong staining).

### Western Blot Analysis

Cells were homogenized in RIPA lysis buffer for 30 minutes and then centrifuged at 14000 ×*g* for 20minutesat 4°C. Western blotting was obtained utilizing 30 µg of whole extract protein according to the standard protocol. Antibodies were used in the study: anti-Fbw7 (for western blots) (ab109617, Abcam, Cambridge, UK), anti-Fbw7 (for IHC) (H00055294-M02, Abnova, Taipei City, Taiwan), anti-LDHA, 3682; anti-phospho-LDHATyr10, 8176; anti-HK2, 2867; anti-Glut1, 12939s; anti-PDK1, 5662; anti-Ubiquitin, 3936; anti-Flag Tag, 14793; anti-His Tag, 9991 (Cell Signaling Technology, Beverly, MA, USA), anti-β-actin, 60008-2-Ig (Proteintech, Rosemont, IL, USA).

### Immunoprecipitation and Ubiquitination Assay

For protein immunoprecipitation analysis, cells were lysed and 1μg of antibodies (anti-Fbw7 or anti-LDHA) was added, which was incubated with protein A/G beads (Millipore) and eluted in SDS buffer at 95°Cfor 4 min. The beads were then washed and boiled in SDS loading buffer and immunoprecipitation complexes were detected by western blotting analysis.

For ubiquitylation assay, cells were transfected with indicated plasmids or siRNA and then treated with MG132 (20 μM). And then cells were harvested, lysed and followed by immunoprecipitation of His-tag LDHA and western blotting to detect His-LDHA ubiquitination.

### Quantification PCR Analysis

For mRNA quantification, total RNAs were isolated using RNAiso(Takara, Shiga, Japan) Plus. And 1 µg of total RNA was used for cDNA synthesis using Prime Script RT Master Mix according to the instructions (Takara). Real-time PCR reactions were run on ABI 7500 PCR system (Applied Biosystems, Carlsbad, CA, USA) using SYBR Premix Ex Taq (Takara). The PCR program consisted of 40 cycles at 95°C for 15 s and 60°C for 34 s. Gene expression was determined relative to β-actin (for mRNA) or U6 (for miRNA) and calculated using the 2^−ΔΔCT^ method. The miDETECT A Track™ miRNA qPCR Kit (RIBOBIO, Guangzhou, China) was used to examine the levels of mature miR-223. The gene-specific primers are listed in **Supplementary Table S2**.

### Animal Model

NOD/SCID mice (4 to 6 weeks) were purchased from the Experimental Animal Tech Co. of Weitonglihua (Beijing, China). Mice were injected with 1 ×10^7^ cells in 100 μl PBS in the left flank. The size of the subcutaneous tumor was measured using calipers twice a week. Tumor volume was calculated as [width × height × depth)/2], where W, H and D represent the width, height and the depth (mm) of the tumor, respectively. These experiments were assessed and approved by the Committee of Care and Use of Laboratory Animals of Guangdong Provincial People’s Hospital.

To investigate whether the production of miR-223 could promote DLBCL tumor growth *in vivo*, hsa-miR-223 agomir(RIBOBIO, Guangzhou, China) (1 nmol in 100 μl PBS, twice a week for 4 weeks) was administered intraperitoneally7 days following SU-DHL-2 cell injection.

### Dual Luciferase Reporter Assays

The targeting sites for miR-223 and the Fbw7 3’-UTR were predicted using the TargetScan algorithm (www.targetscan.org). To validate miR-223-predicted targets, a dual luciferase reporter assay was performed according to the manufacturer (Promega, WI, USA). Cells were transiently transfected with Renilla luciferase vector for 3’-UTR of Fbw7(WT) or mutated miR-223 binding sites. The reporter construct was co-transfected with negative control or miR-223 mimic into the indicated cells for 24 hours. Firefly luciferase was used as an internal control. Cell lysates were prepared and luciferase activity was detected using the Spark multimode microplate reader (TECAN, Mannedorf, Switzerland).

### Statistical Analysis

SPSS 23.0 statistical software (SPSS Inc., Chicago, IL, USA) was used for Statistical analyses. All data are showed as the mean ± SD. Experiments were repeated at least three times. One-way analysis of variance or a two-tailed unpaired Student t test was applied to evaluate the data. A multi-way classification analysis of variance tests was performed to assess data obtained from the CCK8 assays and tumor growth. Differences were known as significant at *, P < 0.05; **, P < 0.01; and ***, P < 0.001. Correlations among Fbw7 and LDHA expression were analyzed with a Spearman rank correlation. P < 0.05 was considered to indicate a significant difference. The relationship between Fbw7 level and LDHA were assessed using the Pearson χ^2^ test. Each assay was performed in triplicate.

## Results

### Fbw7 Impaired LDHA-Mediated Glucose Metabolism Reprogramming in ABC DLBCL

**Figure 1:**
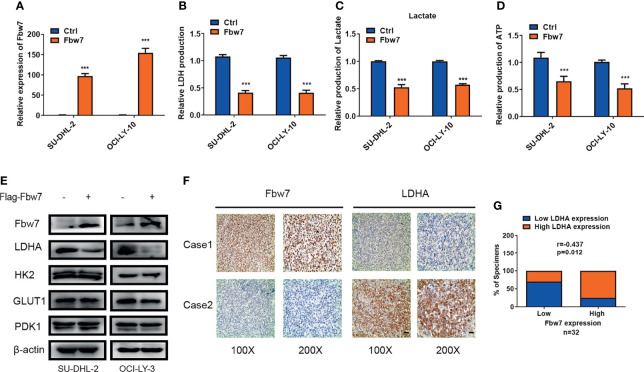
Fbw7 impaired LDHA-mediated glucose metabolism reprogramming in ABC DLBCL. **(A)** QRT-PCR analysis of the relative mRNA expression in OCI-LY-3 and SU-DHL-2 cell lines. Cells were transfected with Flag-Fbw7 and vector control to detect Fbw7 expression. The mean ± SD is shown for three independent experiments. ***P < 0.001; one-way ANOVA. **(B)** Fbw7 inhibited intracellular LDH in ABC-DLBCL cells. The mean ± SD is shown for five independent experiments. ***P < 0.001; one-way ANOVA. **(C)** Fbw7 reduced intracellular lactate production in ABC-DLBCL cells. Results are presented as mean ± SD. ***P < 0.001; one-way ANOVA. **(D)** Fbw7 reduced ATP production. Results are presented as mean ± SD. ***P < 0.001; one-way ANOVA. **(E)** Western blotting results showed that overexpression of Fbw7 mainly inhibited LDHA expression in Glycolysis relative proteins. And the expression of Fbw7, LDHA, HK2, GLUT1, PDK1 were shown. **(F)** IHC staining results of Fbw7 and LDHA were shown with representative images from the same tumor case. The scale bar of “100x” indicates for 100μmand the scale bar of “200x” indicates for 50μm. **(G)** spearman correlation analysis between Fbw7 and LDHA in DLBCL FFPE tissues. Spearman rank correlation. P < 0.05 was considered to indicate a significant difference.

The ubiquitin-ligase Fbw7 plays as a tumor suppressor by targeting lots of proto-oncogenes for proteolysis in cancer. In our early study, we found that Fbw7 significantly regulates apoptosis in ABC-DLBCL (26). To investigate the role of Fbw7 in glucose metabolism reprogramming, we overexpressed Fbw7 in SU-DHL-2 and OCI-LY-3 cells (**Figure 1A**) and then measured intracellular LDH and lactate production which are two basic indicators of the Warburg effect. Results show that Fbw7 reduced intracellular LDH and lactate production significantly, suggesting an inhibitory role of Fbw7 in glycolysis (**Figures 1B, C**). Tumor cells depend on glycolysis for ATP production, which facilitates rapid growth and progression. We analyzed the relationship between Fbw7 expression and ATP production. Expectedly, our study shows that overexpression of Fbw7 inhibited the production of ATP in SU-DHL-2 and OCI-LY-3 cells significantly (**Figure 1D**). To further investigate the role of Fbw7 in glycolysis, the expression of key enzymes of the glycolysis, including GLUT1, HK2, LDHA, and PDK1, were examined. Western blotting analysis revealed that the overexpression of Fbw7 primarily decreased the expression of LDHA compared with other enzymes of glycolysis (**Figure 1E**). To investigate the relationship between Fbw7 and LDHA, 32 samples from DLBCL FFPE tissues were analyzed using IHC analysis. Results showed that higher expressions of Fbw7 were usually accompanied by lower expression of LDHA, while lower expression of Fbw7 was combined with elevated expressions of LDHA in DLBCL tissues. (**Figure 1F**). A Spearman rank correlation analysis furtherly verified that Fbw7 was negatively associated with LDHA (**Figure 1G**). The correlation between Fbw7, LDHA and other clinicopathological features, such as patient DLBCL subtype, age, sex, EB virus status were showed in **Supplementary Table S1**. Taken together, these results show that Fbw7 could impair LDHA-mediated glycolysis in ABC DLBCL cells.

### Fbw7 Interacts With LDHA and Facilitates Its Ubiquitination and Degradation

**Figure 2:**
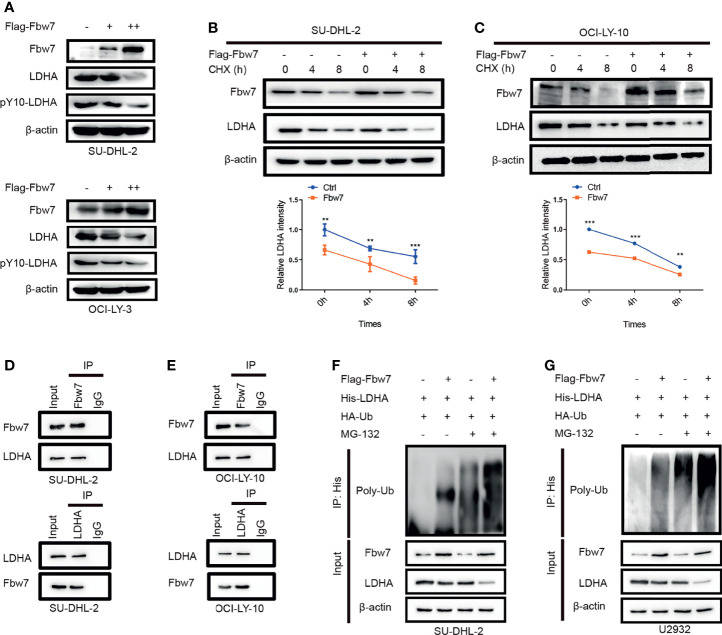
Fbw7 interacts with LDHA and facilitates its ubiquitination and degradation**. (A)** Western blotting analysis SUDHL-2 and OCI-LY-3 cells transfected with Flag-Fbw7 to detect the expression of LDHA and pY10-LDHA. **(B)** SUDHL-2 cells were transfected with Flag-Fbw7 and treated with cycloheximide (CHX; 10 mg/ml) for the indicated time intervals to examine the stability of LDHA. Western blotting results were shown(top) and the mean ratios of the indicated proteins was quantified through densitometry in line graph(bottom). Results are displayed as mean ± SD in the line graph for three independent experiments using; **P < 0.01; ***P < 0.001; one-way ANOVA. **(C)** the similar results were shown in OCI-LY-10 cells. Results are displayed as mean ± SD in the line graph for three independent experiments using; **P < 0.01; ***P < 0.001; one-way ANOVA. **(D, E)**. interaction between endogenous Fbw7 and LDHA in indicated cell lines. Cell lysates from SU-DHL-2 and OCI-LY-10 cells were co-immunoprecipitated with anti-Fbw7 or anti-LDHA antibody and the original results were analyzed by western blotting. IgG was used as a control. **(F, G)** SU-DHL-2 and OCI-LY-10 cells were transfected with the indicated plasmids. Cells were treated with 10 mM MG132 for 6 hours after transfection, the Fbw7-mediated polyubiquitination status of LDHA was examined by immunoblotting.

To identify the molecular mechanism of Fbw7-induced decreased expression of LDHA, we performed western blotting in SU-DHL-2 and OCI-LY-10 cells with increasing amounts of Fbw7 to measure LDHA protein levels. Results showed that overexpression of Fbw7 significantly decreased LDHA expression compared with negative control (**Figure 2A**). Then, we performed cycloheximide (CHX) chase assay to confirm ifFbw7 regulates LDHA activity or stability. After 24 hours post-transfection, SU-DHL-2 cells added with cycloheximide (CHX, an inhibitor of protein synthesis) and the stability of LDHA was detected by western blotting (**Figure 2B** top). Relative LDHA intensity was made by gray analysis and the results show half-life of LDHA reduced from 8 h to approximately 4 h following Fbw7 upregulation (**Figure 2B** bottom). Similarly, upregulation of Fbw7 significantly decreased the stability of LDHA levels in OCI-LY-3 cells (**Figure 2C**). These results indicate that Fbw7 reduced LDHA stability by accelerating its degradation.

We next explored the mechanism by which Fbw7 alters LDHA stability and investigated the interaction of Fbw7 and LDHA. Co-immunoprecipitation analysis demonstrated that Fbw7 interacted with LDHA in SU-DHL-2 cells (**Figure 2D**). Similarly, co-immunoprecipitation of Fbw7 and LDHA were demonstrated in OCI-LY-3 and OCI-LY-10 cells (**Figure 2E**). More importantly, a key phosphorylated tyrosine residue of LDHA phosphorylation sites (Y10) was detected by mass spectrometry of Fbw7 co-immunoprecipitation lysis buffer, which was harvested and analyzed by reversed-phase liquid chromatography-tandem mass spectrometry (LC-MS/MS) (**Figure S1**). These results show that Fbw7 directly interacts with LDHA.As lots of substrate are reported for Fbw7 to recognize and ubiquitination, we furtherly explored whether Fbw7 directly interact with LDHA and facilitates its ubiquitination and degradation. To investigate this possibility, SU-DHL-2 and U2932 cells were transfected with a plasmid encoding His-LDHA and HA-ubiquitin along with and without Flag-Fbw7.Immunoblotting revealed that the level of ubiquitination’s LDHA significantly increased after upregulation of Fbw7 with or without of MG132, an inhibitor of the 26S proteasome (**Figures 2F, G**). Our results imply that Fbw7 interacts with LDHA to facilitate its ubiquitination and degradation.

### Inhibition of LDHA Attenuates Glycolysis and Suppresses Tumor Proliferation in ABC-DLBCL

**Figure 3:**
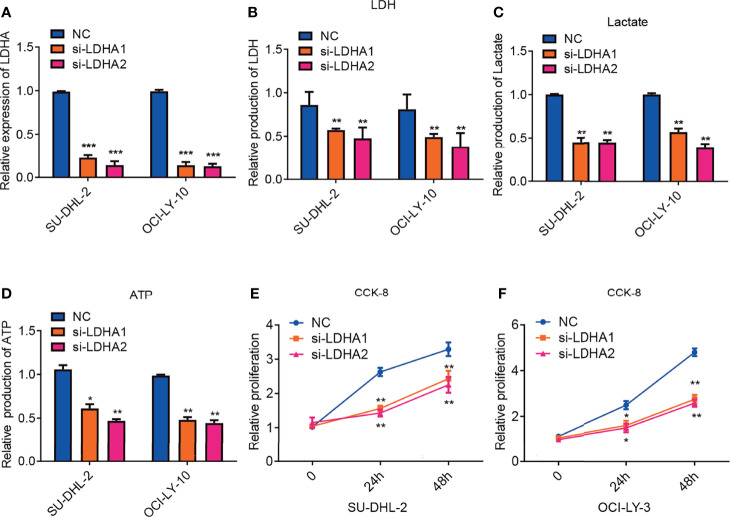
Inhibition of LDHA attenuates glycolysis and suppresses tumor proliferation in ABC-DLBCL. **(A)** QRT-PCR analysis of LDHA mRNA expression in SU-DHL-2 and OCI-LY-10 cell lines. LDHA expression was detected after transfecting with siRNA of LDHA. Experiments were performed in triplicate, and data are presented as the mean ± SD. ***P < 0.001; one-way ANOVA. **(B)** downregulation of LDHA inhibits intracellular LDH in SU-DHL-2 and OCI-LY-10 cells. Experiments were performed in triplicate, and data are presented as the mean ± SD. **P < 0.01; and ***P < 0.001; one-way ANOVA. **(C)** inhibition of LDHA reduced intracellular lactate production in SU-DHL-2 and OCI-LY-10 cells. Experiments were performed in triplicate, and data are presented as the mean ± SD. **P < 0.01; one-way ANOVA. **(D)** inhibition of LDHA reduced ATP production in SU-DHL-2 and OCI-LY-10 cells. Experiments were performed in triplicate, and data are presented as the mean ± SD. *P < 0.05; and **P < 0.01; one-way ANOVA. **(E, F)** CCK8 proliferation assays were examined to determine cell proliferation of in SU-DHL-2 and OCI-LY-10 cells after transfecting with siRNA of LDHA. A multi-way classification analysis of variance tests was performed to assess data obtained from the CCK8 assays and data are shown as mean ± SD in the bar graph for three independent experiments. *P < 0.05; **P < 0.01.

It has been reported that LDHA was pivotal for cell viability and tumor cell proliferation and elevated expression of serum LDH is associated with poor prognosis in DLBCL patients (19, 27). To explore the exact function of LDHA in DLBCL, SU-DHL-2 and OCI-LY-10 cell lines were transfected with normal control or siRNA against LDHA. Quantitative PCR confirmed that LDHA expression was significantly reduced following transfection (**Figure 3A**). We also measured intracellular production of LDH and lactate, two basic indicators of the Warburg effect. Consistently, downregulation of LDHA decreased intracellular the production LDH and lactate (**Figures 3B, C**). Tumor cells rely on glycolysis for energy production which match cell demands for rapid growth and metastasis. We furtherly detected the impact of LDHA on ATP production. As expected, downregulation of LDHA reduced production of ATP in cell lines of SU-DHL-2 and OCI-LY-3 cells (**Figure 3D**). Cell proliferation was confirmed using the Cell Counting Kit-8(CCK-8) assay and the results indicated that silencing LDHA expression significantly inhibited the proliferation of DLBCL cells (**Figures 3E, F**). In conclusion, LDHA is essential for maintaining glycolysis in ABC DLBCL cells, which could influence the proliferation of ABC DLBCL.

### Lactate Decreases Fbw7 Expression in ABC-DLBCL

**Figure 4:**
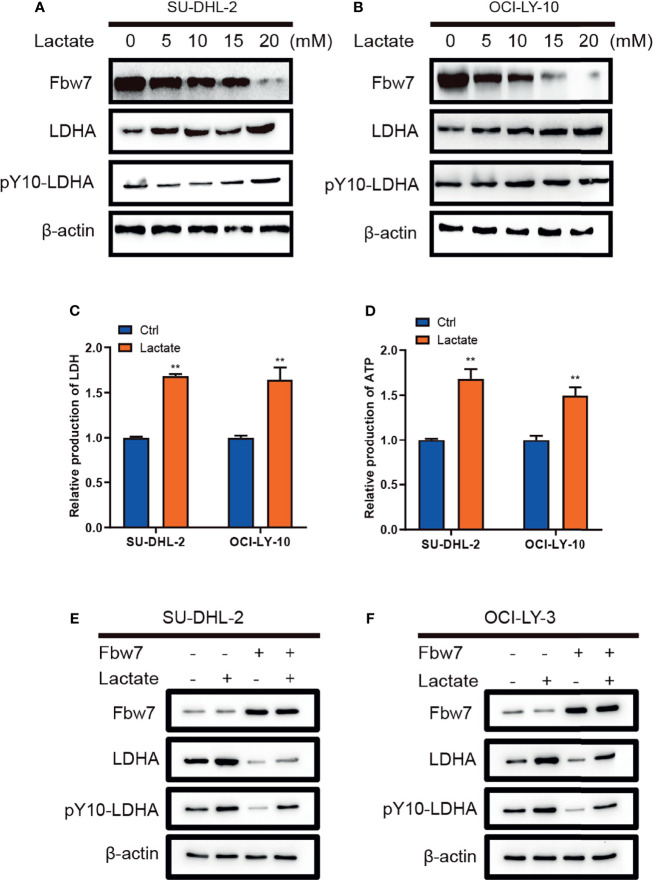
Lactate decreases Fbw7 expression in ABC-DLBCL. **(A, B)** lactate was added into culture medium of SUDHL-2 and OCI-LY-10 cells in a range of concentrations and Fbw7, LDHA, and pY10-LDHA levels were determined by western blotting analysis. **(C)** lactate promotes intracellular LDH in SUDHL-2 and OCI-LY-10 cells. The mean ± SD is shown for five independent experiments. **, P <0.01; one-way ANOVA. **(D)** lactate promotes ATP production SUDHL-2 and OCI-LY-10 cells. The mean ± SD is shown for five independent experiments. **, P <0.01; one-way ANOVA. **(E, F)** western blotting shows the expression of LDHA and pY10-LDHA after transfection of Fbw7 and then addition of lactate.

Lactate as the end product of glycolysis, has normally been deemed to metabolic waste rather than a fuel for tumor cells (28). Interestingly, previously publication had described the metabolism of lactate in human lung tumors *in vivo*, which shows exogenous lactate was consumed and used as a respiratory fuel, predominantly over glucose (29). Therefore, we investigated the role of lactate in DLBCL and its impact on the expression of Fbw7 and LDHA. Lactate was added into culture medium of SU-DHL-2 and OCI-LY-10 cells in a range of concentrations and Fbw7, LDHA, and pY10-LDHA levels were determined by western blotting analysis. The results showed that lactate inhibited the expression of Fbw7 and enhanced LDHA and pY10-LDHA expression with a positive feedback effect (**Figures 4A, B**). Next, we measured intracellular LDH and lactate in DLBCL cells. Consistently, lactate increased the production of intracellular LDH and lactate production (**Figures 4C, D**). To investigate the effect of lactate in the Fbw7-mediated reduction of LDHA, we treated cells with lactate to measure the expression of Fbw7 in SU-DHL-2 and OCI-LY-10 cells. Western blotting analysis showed that the supplementary lactate significantly increased the expression of LDHA and pY10-LDHA and reversed the Fbw7-mediated inhibition of LDHA (**Figures 4E, F**). Taken together, our data indicate that lactate attenuates Fbw7 expression to form a negative feedback loop.

### MiR-223 Triggered by Lactate Targets Fbw7 and Promoted DLBCL Proliferation

As stated above, we found that lactate could inhibit the expression of Fbw7 and promotes the expression of LDHA and pY10-LDHA in SU-DHL-2 and OCI-LY-10 cells. However, the underlying mechanism by which lactate suppresses Fbw7 in DLBCL is unknown. Under hypoxic conditions, cancer cells may exhibit an increased dependency on glycolysis compared with normal tissues. LDHA catalyzes pyruvate to lactate resulting in the production of large molecular compounds that are required for a wide range of cellular processes including ATP production.

**Figure 5:**
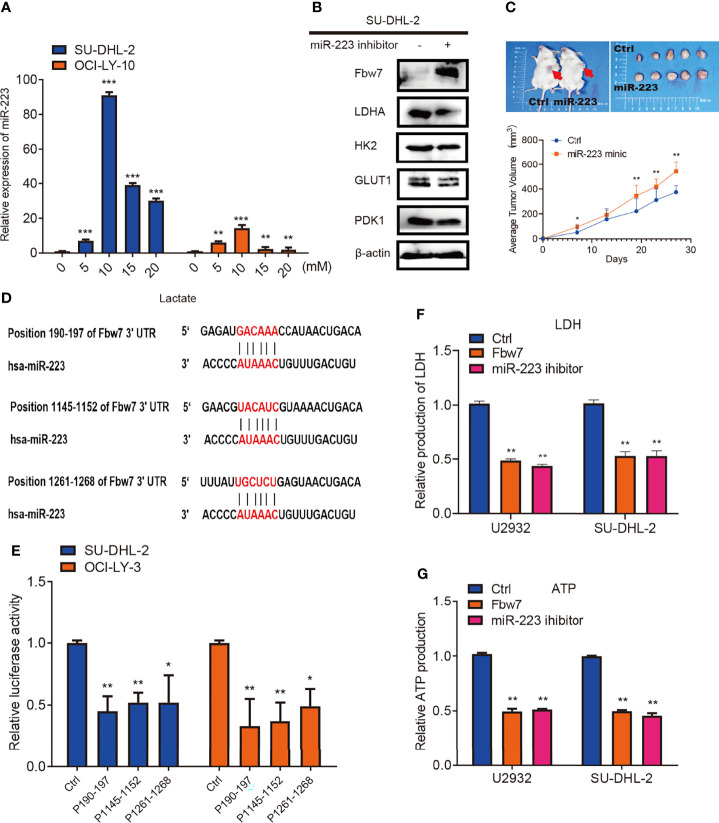
miR-223 triggered by lactate targets Fbw7 and promoted DLBCL proliferation. **(A)** qPCR analysis of the relative miR-223 expression in SU-DHL-2 and OCI-LY-3 cell lines. Lactate was treated into cells with the indicated concentration gradient. The mean ± SD is shown for five independent experiments. **P < 0.01. ***P < 0.001; one-way ANOVA. **(B)** western blots showed miR-223 inhibitor increases Fbw7 expression and mainly reduces LDHA expression in Glycolysis relative proteins. And the expression of Fbw7, LDHA, HK2, GLUT1, PDK1 were shown. **(C)** miR-223 mimic promotes SU-DHL-2 cell proliferation *in vivo*. Representative images of tumors proliferation in NOD-SCID mice. Mice were implanted with 1 × 10^7^ cells into the flanks of mice (n = 5). Here, mice were treated with miR-223 agomir and agomir of normal control twice a week. 27 days after tumor implantation, mice were sacrificed and tumor volume was measured. 1 cm for Scale bar. A multi-way classification analysis of variance tests was performed to assess data obtained from the tumor volume assays and data are shown as mean ± SD. P < 0.01. **(D)** binding sites of miR-223 and the 3’-UTR of Fbw7 were shown for three conserved sites which was determined by the TargetScan algorithm. **(E)** reporter constructs containing a position of Fbw7 190-197 3’-UTR region (wild-type, WT) or Fbw7 (MUT) with mutated miR-223 binding site of were co-transfected with a control oligo (mimic ctrl) or miR-223 mimic oligo into HEK293 cells. Luciferase activity was detected 24 hours after transfection. Data are shown as mean ± SD; n=5. *P < 0.01; **P < 0.01; one-way ANOVA. **(F)** Fbw7 and miR-223 inhibitor reduces intracellular LDH in U2932 and SU-DHL-2 cells. The mean ± SD is shown for five independent experiments. **P < 0.01; one-way ANOVA. **(G)** Fbw7 and miR-223 inhibitor reduced ATP production in U2932 and SU-DHL-2 cells. The mean ± SD is shown for five independent experiments. **P < 0.01; one-way ANOVA.

MicroRNAs (miRNAs), a class of noncoding endogenous RNAs with 18-24 nt length, which participate in the post-transcriptional regulation of eukaryotic genes (30). MiRNAs are involved in the control of a multiplicity of biological processes, and their absence or altered expression has been associated with a variety of human diseases, including cancer (31). Recent studies show that miR-223 is either endogenously expressed or transferred in exosomes or extracellular vesicles to non-phagocytic cells including cancer cells, where it exerts biological functions. In cancerous cells, miR-223 acts either as an oncomiR promoting tumors or as a tumor suppressor in a context-dependent manner (32). It was previously reported that the miR-223/Fbw7 axis plays an important role in lung cancer cells under hypoxic conditions and miR-223 has been shown to target Fbw7 in various cancers (33–38). Hence, we further investigated whether lactate inhibits Fbw7 *via* altering the expression of miR-223. Lactate was added to cells in concentrations ranging from 0 to 20 mM. Quantitative PCR analysis revealed that the expression of miR-223 significantly increased after lactate treatment (**Figure 5A**). To further elucidate the relationship between miR-223 andFbw7 in DLBCL, we treated DLBCL cells with miR-223 inhibitors. Western blotting analysis revealed a significant increase in Fbw7 expression and decreased LDHA expression among the glycolysis-related enzymes (**Figure 5B**). To investigate the role of miR-223 in DLBCL progression *in vivo*, NOD-SCID mice were injected with SU-DHL-2 cells and then treated with miR-223 mimics twice a week. In this vivo model, cells treated with miR-223 mimics promoted tumor growth (**Figure 5C**). Moreover, three conserved binding sites for miR-223 and the Fbw7 3’-UTR were identified by the TargetScan algorithm (**Figure 5D**). To confirm the effect of miR-223 targeting Fbw7 in DLBCL, we measured the luciferase activity of the 3’-UTR region (190-197) of Fbw7 (WT-3’-UTR) and its mutant. The wild type 3’-UTR reporter was significantly decreased compared with the mutant 3’-UTR reporter (**Figure 5E**). And the metabolic analysis revealed that Fbw7 and miR-223 inhibitor similarly reduced intracellular LDH and ATP production (**Figures 5F, G**). In conclusion, our data confirmed that lactate-induced miR-223 targets Fbw7 and promotes DLBCL proliferation.

### Inverse Correlation Between Fbw7 and LDHA/Lactate/miR-223 Axis in ABC-DLBCL

**Figure 6:**
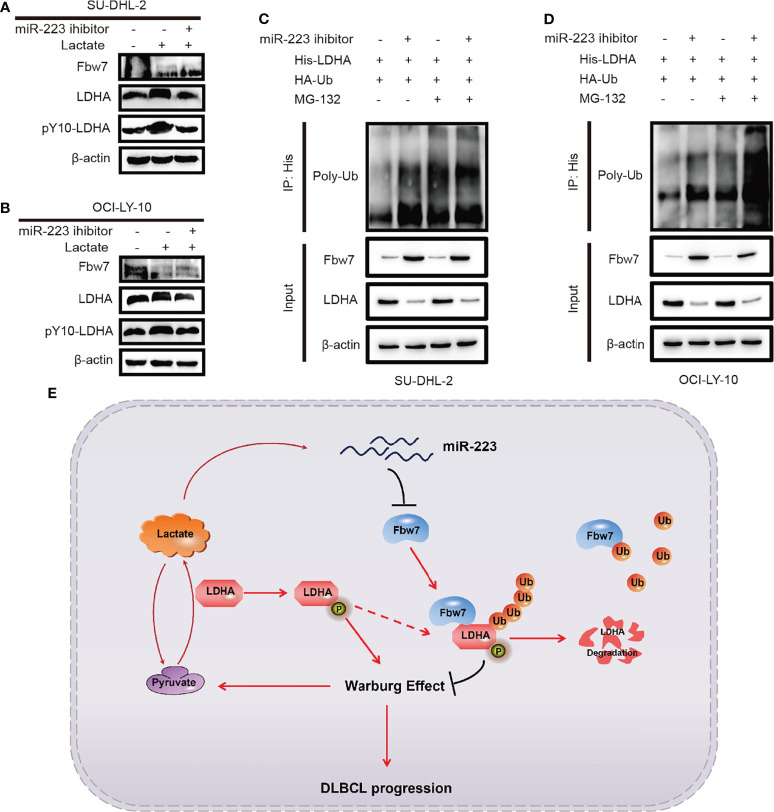
Inverse correlation between Fbw7 and LDHA/lactate/miR-223 axis in ABC DLBCL. **(A, B)** the effects of lactate induction after miR-223 inhibitor was treated in SU-DHL-2 and OCI-LY-10 cells. The expressions of Fbw7, LDHA, pY10-LDHA were examined by western blotting. **(C, D)** His-LDHA and HA-ubiquitin were treated with miR-223 inhibitor in SU-DHL-2 and OCI-LY-10 cells. After treated with 10 mM MG132 for 6 hours, LDHA was co-immunoprecipitated with anti-His antibody, and the polyubiquitination status of LDHA was examined by immunoblotting. **(E)** schematic graph of potential mechanism of Fbw7 negatively regulates the positive feedback loop of the LDHA/lactate/miR-223 in ABC DLBCL.

To investigate the effect of lactate and miR-223 in the Fbw7-medicated reduction of LDHA, SUDHL-2 and OCI-LY-10 cells were treated with lactate and the miR-223 inhibitor. Western blot analysis results revealed that the addition of lactate significantly increased the expression of LDHA and pY10-LDHA. While miR-223 inhibitor reversed the expression of LDHA (**Figures 6A, B**). We previously demonstrate that Fbw7 targeted LDHA for ubiquitination and degradation. Therefore, we hypothesized that miR-223 may reverse the degradation of LDHA. To test this concept, His-LDHA and HA-ubiquitin were co-expressed with and without the miR-223 inhibitor in SU-DHL-2and OCI-LY-10 cells. Co-immunoblotting revealed that the ubiquitination of LDHA increased significantly after cell transfected with miR-223 inhibitors in the presence of MG132 (**Figures 6C, D**). Taken together, these results indicate an inverse correlation between Fbw7 and LDHA/lactate/miR-223 axis in ABC-DLBCL, which may constitute a promising therapeutic target for ABC-DLBCL patients.

## Discussion

Fbw7 act as a ubiquitin ligase and inhibitor of malignant tumors by targeting several key oncogenes for proteolysis. We previously reported that Fbw7 promotes apoptosis in DLBCL. However, the underlying mechanism through which Fbw7 regulates tumor metabolism reprograming remains unknown. Here, we explored the function of Fbw7 in regulating aerobic glycolysis in ABC-DLBCL and revealed that Fbw7 targets LDHA for ubiquitylation and degradation. We also identified a negative functional loop consisting of an inverse correlation between Fbw7 and LDHA/lactate/miR-223 axis in ABC-DLBCL (**Figure 6E**).

The reprogramming of glucose metabolism is pivotal for tumor progression (39). The Warburg effect has been known as an important feature of tumor and a method of ATP generation even under normal oxygen concentrations (40). Fbw7 may regulate a broad range of metabolic pathways *via* targeting several pivotal substrates including HIF-1alpha,c-Myc, SREBP, mTOR, PGC-1α, and REV-ERBα (41). However, the Fbw7-related glucose metabolism reprogramming in DLBCL remains unclear. Firstly, we demonstrated Fbw7 plays a vital role in ABC-DLBCL cell glycolysis by decreasing expression of LDHA protein. Consistently, overexpression of Fbw7 reduced intracellular LDH, lactate, and ATP production, thus demonstrating its inhibitory role in glycolysis. Fbw7 exogenous expression primarily decreased LDHA expression compared with other glycolysis-related enzymes. In addition, IHC staining results revealed that high expression level of Fbw7 was associated with low expression of LDHA in DLBCL FFPE tissues. inversely, lower expression of Fbw7 was combined with elevated expressions of LDHA in DLBCL tissues.

LDHA catalyzes pyruvate to lactate and is known as a vital checkpoint of anaerobic glycolysis. It has been reported that LDHA is upregulated by both HIF-1alpha and Myc in cancer cells to promote lactate production. Fbw7 is capable of governing several key substrates including HIF-1alpha and c-Myc. However, if Fbw7 interact with LDHA directly remains unclear. Our results demonstrated that Fbw7 interacts with LDHA and facilitates its ubiquitination and degradation. Firstly, we found that Fbw7 attenuated the stability of LDHA in a dose-dependent manner and the half-life of LDHA was decreased. Moreover, co-immunoprecipitation experiments showed that Fbw7 interacts with LDHA and the ubiquitination of LDHA was significantly increased following Fbw7 overexpression. Moreover, it’s reported that multisite-phosphorylated substrate recognition by ubiquitin ligases of Fbw7 complex (42, 43). And the key phosphorylated tyrosine residue of LDHA phosphorylation sites (Y10) was detected by mass spectrometry of Fbw7 co-immunoprecipitation lysis buffer using liquid chromatography-tandem mass spectrometry (LC-MS/MS). So Fbw7 targets LDHA for ubiquitylation and degradation may focus on phosphorylated tyrosine residue of LDHA phosphorylation sites (Y10).

Tumors often exhibit increased dependency on glycolysis as their enhanced glucose uptake and preferential use of glycolysis as opposed to oxidative phosphorylation for ATP production (44). Elevated LDHA expression is also associated with poor outcome in tumor patients. Our data clarified that inhibition of LDHA attenuates glycolysis and suppresses tumor proliferation in ABC-DLBCL.

Traditionally, lactate has been considered to be metabolic waste rather than energy source for tumor cells. Interestingly, we found that lactate promotes the expression of LDH and ATP production in ABC DLBCL. More importantly, we demonstrated that lactate promotes LDHA expression in a positive feedback loop. However, the expression of Fbw7 was decreased in lactate supplemented ABC DLBCL cells.

It was reported that the miR-223 suppressed Fbw7 expression in lung cancer cells under hypoxic conditions. In this study, we confirmed that lactate inhibits Fbw7 expression *via* triggering miR-223 even under normal oxygen concentrations. In mechanism, miR-223 targets the 3’-UTR region of Fbw7 to inhibit its expression. Collectively, these results indicated that miR-223, which is induced by lactate, targets Fbw7 and promotes DLBCL proliferation.

Fbw7 is deeply involved in cell development or tumor progression (45–47). In this study, we revealed Fbw7 interacts with LDHA which restraining the positive feedback of the LDHA/lactate/miR-223 axis in ABC DLBCL. Taken together, our results identified the key role of Fbw7 in LDHA-mediated glucose metabolism reprogramming which may serve as a therapeutic target in ABC DLBCL.

## Data Availability Statement

The original contributions presented in the study are included in the article/**Supplementary Material**. Further inquiries can be directed to the corresponding author.

## Ethics Statement

The studies involving human participants were reviewed and approved by Research Ethics Committee of Guangdong Provincial People’s Hospital. The patients/participants provided their written informed consent to participate in this study. The animal study was reviewed and approved by Research Ethics Committee of Guangdong Provincial People’s Hospital.

## Author Contributions

YL and SY carried out the conception and design. TG, FZ, YC, WC, FX, DLL, XL, and ZL participated in the acquisition of data (acquired and managed patients, provided facilities etc.). Cell and molecule experiments: SY, TG, and DYL carried out the cell and molecule experiments. TG carried out the animal experiments. SY and TG carried out the analysis and interpretation of data (statistical analysis, biostatistics, computational analysis). YL drafted the manuscript. Study supervision: YL and SY. All authors have approved the final manuscript.

## Funding

This work was supported by Natural Science Foundation of Guangdong Province (No. 2019A1515011643); National Natural Science Foundation of China (No. 81702322); High-level Hospital Construction Project of Guangdong Provincial People’s Hospital (No. DFJH201914); Medical Science and Technology Research Fund of Guangdong Province of China (No.B2021299).

## Conflict of Interest

The authors declare that the research was conducted in the absence of any commercial or financial relationships that could be construed as a potential conflict of interest.

## Publisher’s Note

All claims expressed in this article are solely those of the authors and do not necessarily represent those of their affiliated organizations, or those of the publisher, the editors and the reviewers. Any product that may be evaluated in this article, or claim that may be made by its manufacturer, is not guaranteed or endorsed by the publisher.

## Abbreviations

Fbw7, F-box and WD repeat domain-containing 7; DLBCL, diffuse large B-cell lymphoma; LDHA, Lactate Dehydrogenase A; ABC, activated B-cell; GCB, germinal center B cell; LDH, lactate dehydrogenase; IPI, International Prognostic index; STR, short tandem repeats; ATCC, American Type Culture Collection; IHC, immunohistochemistry; CCK8, Cell Counting Kit-8.
