# Supplementary material for: Fbw7 Inhibits the Progression of Activated B-Cell Like Diffuse Large B-Cell Lymphoma by Targeting the Positive Feedback Loop of the LDHA/lactate/miR-223 Axis
Source: Front Oncol. 2022 Mar 10;12:842356. doi: 10.3389/fonc.2022.842356 (PMC8960958; doi:10.3389/fonc.2022.842356)
Supplement: Supplementary file 1 [file DataSheet_1.pdf]

Supplementary Fig S1.

a

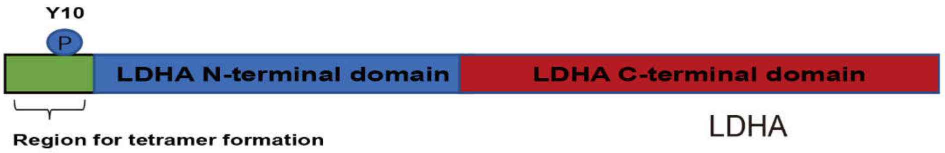

b

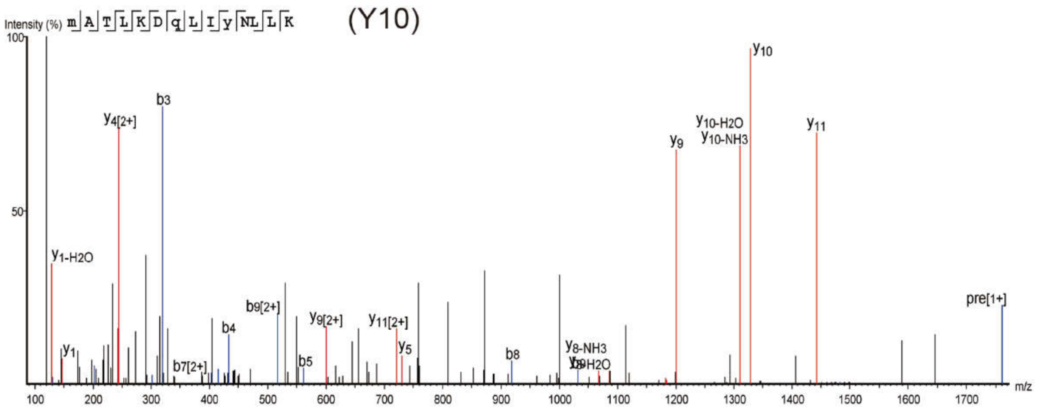

Supplementary Figure S1. Fbw7 targets LDHA with a key phosphorylated tyrosine residue of phosphorylation sites (Y10) for ubiquitination and proteasomal degradation. a, the schematic representation of LDHA is shown with a key phosphorylated tyrosine residue of phosphorylation sites (Y10). b, by mass spectrometry of Fbw7 co-immunoprecipitation samples, Y10 phosphorylation of LDHA was detected. The resulting peptides were harvested and analyzed by reversed-phase liquid chromatography-tandem mass spectrometry (LC-MS/MS).

Supplementary Fig S2.

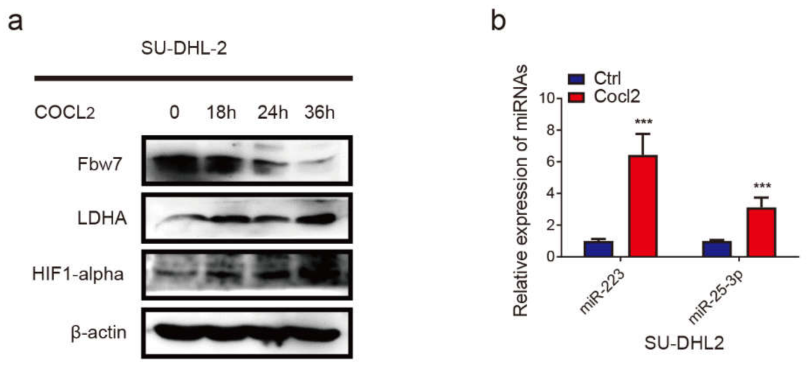

Supplementary Figure S2. Hypoxic Induction miRNAs targets Fbw7 in DLBCL. a, western blots showed hypoxic induction of Cocl2 inhibits Fbw7 expression and the expression of LDHA, HIF1-alpha were shown. b, qPCR analysis two pivotal miRNAs of miR-223 and miR-25-3p expression in SU-DHL-2 cell lines under hypoxic induction of Cocl2, which were reported for targeting Fbw7. The mean ± SD is shown for five independent experiments. ***, $P < 0.001$.
