# Supplementary material for: Fbw7 Inhibits the Progression of Activated B-Cell Like Diffuse Large B-Cell Lymphoma by Targeting the Positive Feedback Loop of the LDHA/lactate/miR-223 Axis
Source: Front Oncol. 2022 Mar 10;12:842356. doi: 10.3389/fonc.2022.842356 (PMC8960958; doi:10.3389/fonc.2022.842356)
Supplement: Supplementary file 2 [file Table_1.doc]

**Supplementary Table S1. Correlation between LDHA expression and clinicopathological variables in 32 DLBCL cases**

| **Characteristics** | **All cases(N=32)** |  | **LDHA Expression (%)** | | |
| --- | --- | --- | --- | --- | --- |
|  |  | **Low (n=15)** | **High (n=17)** | **χ2 value** | ***p* value** |
| **DLBCL substype** |  |  |  | 0.974 | 0.332 |
| GCB | 10 | 6 | 4 |  |  |
| Non-GCB | 22 | 4 | 13 |  |  |
| **Age (years)** |  |  |  | 0.241 | 0.627 |
| ≤50 | 8 | 3 | 5 |  |  |
| >50 | 24 | 12 | 12 |  |  |
| **Sex** |  |  |  | 0.071 | 0.492 |
| Man | 20 | 9 | 11 |  |  |
| Woman | 12 | 6 | 6 |  |  |
| **EB virus** |  |  |  | 1.875 | 0.181 |
| Negative | 30 | 15 | 15 |  |  |
| Positive | 2 | 0 | 2 |  |  |
| **Serum LDH** |  |  |  | 0.511 | 0.480 |
| Normal | 17 | 9 | 8 |  |  |
| Abnormal | 15 | 6 | 9 |  |  |
| **Fbw7 expression** |  |  |  | 7.064 | 0.012 |
| Low | 20 | 6 | 14 |  |  |
| High | 12 | 9 | 3 |  |  |

**Supplemental Table S2. Primers for quantitative PCR**

| Primer | Forward (5’-3’) | Reverse (5’-3’) |
| --- | --- | --- |
| Fbw7 | ACTGGGCTTGTACCATGTTCA | TGAGGTCCCCAAAAGTTGTTG |
| LDHA | ATGGCAACTCTAAAGGATCAGC | CAACCCCAACAACTGTAATCT |
| β-actin | CATGTACGTTGCTATCCAGGC | CTCCTTAATGTCACGCACGAT |
